# Serum Immunoglobulin G Antibodies to Human Papillomavirus Type 6 L1, E2, E4, E6, and E7 Proteins Among Children Prospectively Followed up for 3 Years

**DOI:** 10.1093/infdis/jiae293

**Published:** 2024-05-31

**Authors:** Helmi Suominen, Kari Syrjänen, Tim Waterboer, Seija Grénman, Stina Syrjänen, Karolina Louvanto

**Affiliations:** Department of Obstetrics and Gynecology, Tampere University, Tampere; SMW Consultants, Ltd, Kaarina, Finland; Division of Infections and Cancer Epidemiology, German Cancer Research Center, Heidelberg, Germany; Department of Obstetrics and Gynecology, Turku University Hospital, Turku; Department of Oral Pathology and Oral Radiology, University of Turku, Turku; Department of Obstetrics and Gynecology, Turku University Hospital, Turku; Department of Oral Pathology and Oral Radiology, University of Turku, Turku; Department of Obstetrics and Gynecology, Tampere University, Tampere; Department of Oral Pathology and Oral Radiology, University of Turku, Turku; Department of Obstetrics and Gynecology, Tampere University Hospital, Tampere, Finland

**Keywords:** human papillomavirus, HPV-6, IgG antibody, serology, seroconversion

## Abstract

**Background:**

Current knowledge implicates that human papillomavirus (HPV) infection can be acquired at an early age. However, the role of HPV-specific passive immunization from mother to neonate is nearly unexplored, especially against the HPV early proteins. We analyzed immunoglobulin G (IgG) antibodies against HPV-6 early (E2, E4, E6, E7) and late (L1) proteins in children prospectively followed up for 3 years.

**Methods:**

A total of 272 children and their mothers from the Finnish Family HPV Study were included in these analyses. Serum samples were obtained from pregnant mothers at their third trimester and from newborn/infants at 1-, 2-, 6-, 12-, 24-, and 36-month visits after birth. Antibodies were analyzed by multiplex serology based on glutathione S-transferase fusion protein capture to fluorescent beads.

**Results:**

Maternal antibodies to all tested HPV-6 proteins were transferred to neonates, concordance between maternal and neonates’ antibody levels being highly significant (*P* < .001). Seropositivity of HPV-6 L1 in the neonates declined during the first 6 months of life, whereas changes in the E protein antibodies were less obvious. After the maternal antibodies had vanished, seroconversion to HPV-6 L1 at 12 months (median) and to the HPV-6 E proteins between 23 and 35 months was observed.

**Conclusions:**

IgG antibodies against HPV-6 E and L proteins are transferred from mothers to their children. Seroconversion against HPV-6 L1, E2, E4, E6, and E7 does occur in early childhood, as a sign of acquired HPV-6 infection by vertical or horizontal transmission starting at 12 months of age.

## Teaser

Maternal antibodies against human papillomavirus type 6 (HPV-6) early and late proteins are transferred to their offspring, and our results demonstrate that seroconversion to HPV-6 L1, E2, E4, E6, and E7 does occur in early childhood.

Human papillomavirus (HPV) can be transmitted both sexually and nonsexually. Nonsexual transmission can occur by vertical transmission from the mother to neonate, horizontal transmission from close contact among family members or others, and indirect transmission via contaminated objects [1–8]. Accordingly, HPV DNA has been detected in the placenta, amniotic fluid, umbilical cord, fetal membranes, blood, sperm, and genital tract [3, 4, 9, 10]. Vertical transmission of HPV could potentially occur periconceptually (during fertilization), prenatally (during pregnancy), or perinatally (during birth or immediately afterward) [3, 4, 9, 10]. During pregnancy, passive immunization does occur, when maternal IgG antibodies are transferred via placenta to the fetus to protect the neonate against infection [11, 12]. An active transplacental immunoglobulin G (IgG) transfer starts at 13 weeks and peaks in the last trimester, when the majority of IgG is transferred during the final 4 weeks of gestation [12]. Thus, maternal IgG antibodies are designed to protect against HPV infection during the fetal and/or neonatal period. One important factor affecting the neonatal immunity is the concentration of these maternal antibodies [13].

The replication cycle of HPV is divided into early and late phases. Viral replication factor E1 and its auxiliary protein E2 are probably the first proteins expressed during the infection [14, 15]. E6 and E7 genes are also expressed during early infection but at a lower level. They stimulate the cell cycle progression in differentiating epithelial cells and allow HPV-infected cells to survive and replicate. E4 and E5 are expressed at highest levels in differentiating virus-infected cells and are required for productive viral genome amplification. E4 has also been suggested to be part of the virion release and contribute to the viral transmission [16]. The late proteins L1 and L2 are expressed always subsequent to E4 protein [17, 18]. Besides having individual functions in the HPV viral life cycle, it has been suggested that the viral proteins including both HPV early and late proteins interact with each other and therefore these protein complexes may have additional functions beyond those of the individual proteins [15].

The role of passive immunization in vertical transmission of HPV infection is nearly unexplored, because only a few studies have explored HPV serology in neonates, young children, and their mothers [4, 19–22], nearly all being focused only on HPV L1 serology. Considering the increasing coverage of prophylactic HPV vaccination among future mothers, maternal antibodies against HPV and their impact on children are of great interest. First, serological response to HPV-6 early (E) proteins in infants and young children would provide additional evidence for HPV-6 transmission at an early age. Furthermore, immune response against HPV-6 is clinically important, because this HPV genotype causes the majority of juvenile-onset laryngeal papillomas, which are recalcitrant to treat in neonates and young children. In the present study, we analyzed serum IgG antibodies to HPV-6 L1 protein as well as to HPV-6 early proteins E2, E4, E6, and E7 among neonates prospectively followed up for 3 years.

## MATERIALS AND METHODS

### Participants

The Finnish Family HPV (FFHPV) Study is a longitudinal cohort study run since 1998 and designed to investigate the transmission of HPV within regular Finnish families. The FFHPV study is jointly run by the Department of Obstetrics and Gynecology, Turku University Hospital and the Institute of Dentistry, Faculty of Medicine, University of Turku. The study protocol was originally approved by the Research Ethics Committee of Turku University Hospital (#3/1998), with subsequent amendments in 2006 and 2010 (#2/2006 and 45/180/2010). Altogether, 329 mothers, 131 fathers, and 331 newborns were enrolled in the study between 1998 and 2001. None of the participants had received a prophylactic HPV vaccination, and therefore all measured antibodies result from a naturally acquired HPV infection. The child's health at the age of 1 and 3 years was surveyed using a structured questionnaire addressed to the parents.

Written informed consent was obtained from all participants, whose characteristics have been described elsewhere [23–26].

### Samples and Serological Analysis

Serum samples were obtained from the mothers at their third trimester of pregnancy, and from their offspring at 6 follow-up visits: 1 month (n = 232), 2 months (n = 239), 6 months (n = 263), 12 months (n = 272), 24 months (n = 250), and 36 months (n = 243) visits after birth. The blood samples were centrifuged at 1150*g* for 10 minutes (Sorvall GLC-2, DuPont Instruments), and the serum was divided into three 1-mL aliquots and stored first at −20°C for no longer than a week and then at −70°C until analysis at the German Cancer Research Center (DKFZ), Heidelberg, Germany. Antibodies to HPV-6 E2, E4, E6, E7, and late (L) protein L1 were analyzed by multiplex serology based on glutathione S-transferase fusion protein capture to fluorescent beads [27]. Further details on the sample collection, validation, and their processing at the DKFZ have been described previously [23, 26]. Cut-off value of seropositivity was median fluorescence intensity (MFI) ≥100 for E2, E4, E6, and E7, and ≥200 for L1.

### Statistical Analyses

Differences in the means of continuous variables were analyzed using Mann-Whitney test or Kruskal-Wallis test for 2 and multiple independent samples, respectively. Mean HPV-6 L1 antibody levels were compared to those of HPV-6 E2, E4, E6, and E7 using Pearson *R* correlation coefficient. Seroconversion was defined if at least a 2-fold increase from the previous MFI value was found and the new MFI value exceeded the cut-off level. Correlations between (1) HPV-6 E2, E4, E6, E7, and L1 antibody levels in the baseline samples of the mothers and (2) those of their children at the age of 1 month were analyzed using the Spearman *R* correlation coefficient. The same test was used to analyze correlations between HPV-6 L1 and individual E2–E7 antibody levels of the neonate/child at each follow-up visit. All analyses were done using SPSS for Windows, version 26.0 (IBM SPSS Statistics for Windows, Armonk, New York) and Stata (Stata/SE 14.1, Stata Corp, College Station, Texas). All statistical tests performed were 2-sided and declared significant at *P* < .05.

## RESULTS

**Figure 1.**
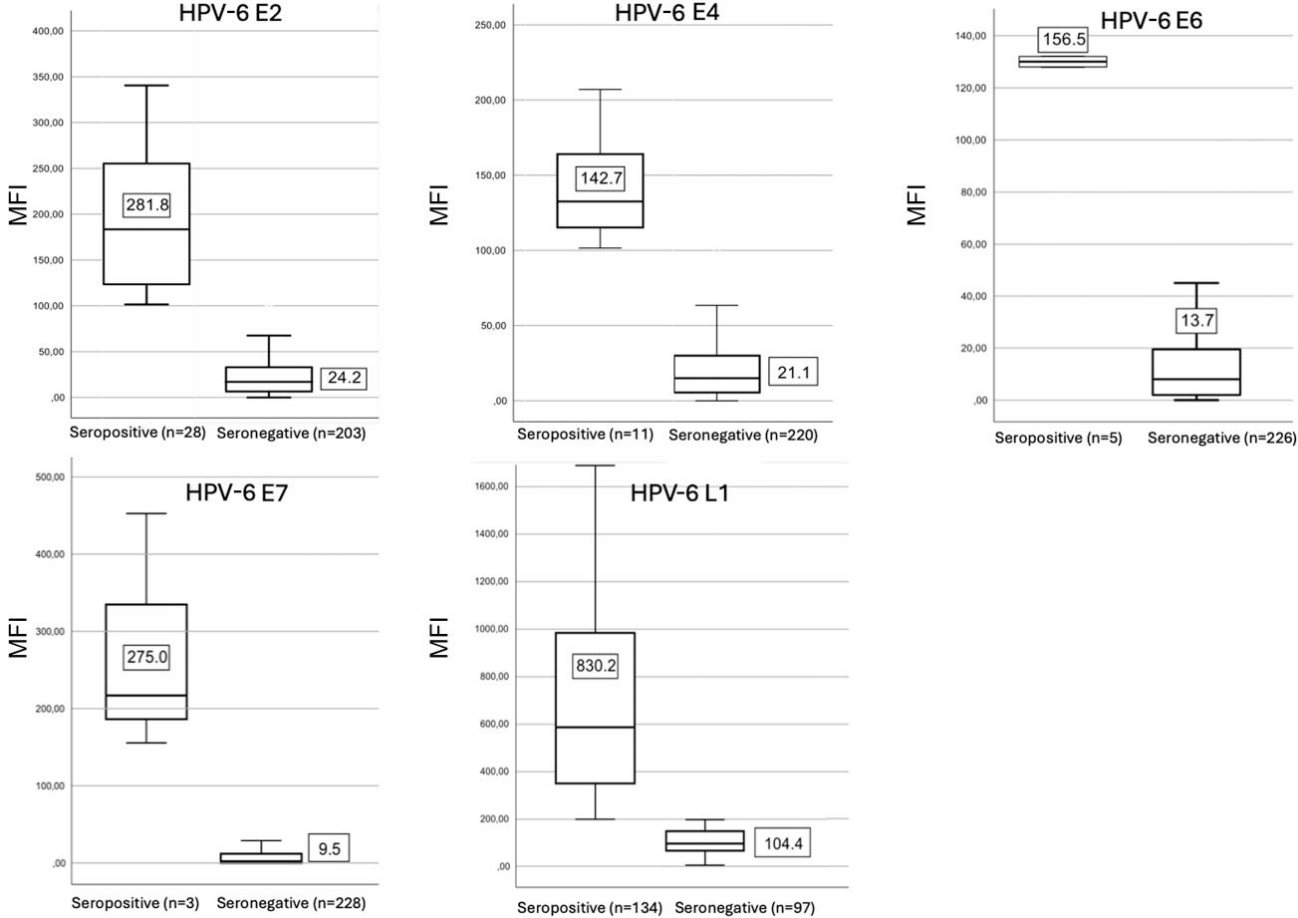
Human papillomavirus type 6 (HPV-6) E2, E4, E6, E7, and L1 antibody levels (median fluorescence intensity [MFI], mean ± standard deviation) among pregnant mothers in their third trimester at the baseline of the study, separately for seropositive and seronegative women. Cut-off for L1 seropositivity was MFI ≥200. Cut-off for E2, E4, E6, and E7 seropositivity was MFI ≥100. Abbreviation: n = number of the mothers stratified by their human papillomavirus serostatus.

The mean antibody levels to HPV-6 E2, E4, E6, E7 and L1 in the baseline samples (third trimester of pregnancy) of all expectant mothers as stratified by their serostatus (+/−) are shown in Figure 1. In both groups, the highest mean MFI values were found for HPV-6 L1 antibodies—830 and 104 in seropositive and seronegative mothers, respectively. Of the E protein antibodies, the highest mean MFI values were found to E2 (MFI = 282) and E7 (MFI = 275). Antibody levels to HPV-6 E4 and E6 were the lowest (MFI values were 143 and 157, respectively).

In Table 1, the antibody titers in the mothers’ baseline samples are correlated with those of their neonate at the age of 1 month. Maternal antibodies to both HPV-6 E and L1 proteins were significantly correlated with the respective antibody titers in the neonate (*P* = .001 for all). At the age of 12 months, the infants’ HPV-6 antibodies had lost their significant correlation with the baseline maternal antibody levels.

**Table 1.** Correlation Between Maternal Human Papillomavirus Type 6 L1, E2, E4, E6, and E7 Antibody Levels at Baseline and Those of the Neonate Sampled at the 1-Month Follow-up Visit

HPV-6 Protein	Pearson Correlation Coefficient (*r*)	Significance (*P* Value)
L1	0.854	.001
E2	0.640	.001
E4	0.679	.001
E6	0.546	.001
E7	0.562	.001

Abbreviation: HPV-6, human papillomavirus type 6.

**Figure 2.**
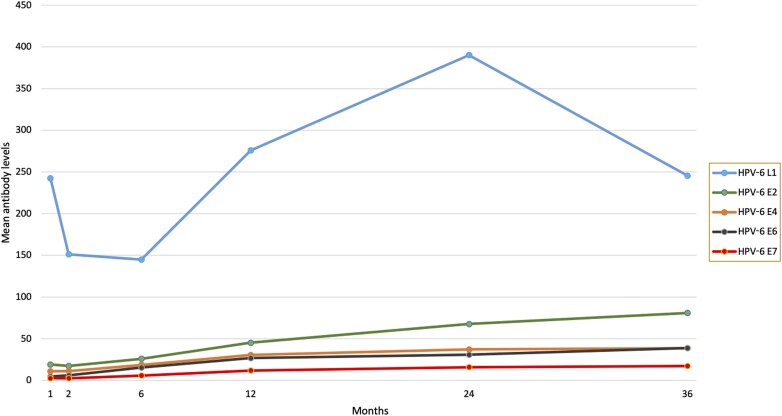
Human papillomavirus type 6 (HPV-6) E2, E4, E6, E7, and L1 antibody levels (mean MFI) at each follow-up visit of the infants.

The graphs in Figure 2 summarize the levels of HPV-6 E and L1 antibodies during the first 3 years of life in the children. In this longitudinal setting, the pattern of HPV-6 L1 antibodies is different from that of the HPV-6 E antibodies in 2 respects: (1) the L1 antibody titers are of completely different magnitude as all the others; and (2) a distinct decline of the titer during the first 6 months was shown, followed by a deep increase between month 6 and 24 (peaking), and decline thereafter. The mean levels of HPV-6 E antibodies follow a less pronounced pattern, characterized by a stable state until month 6 and slight but steady increase thereafter. Of the E-protein antibodies, the increase of mean levels seems to be most pronounced for E2 antibodies.

Table 2 depicts the IgG antibody (mean ± standard deviation) levels to HPV-6 L1 and E proteins among newborns and children during the follow-up. Indicated are also the correlations between L1 antibody levels and those of the E protein antibodies (significant correlations bolded). In Pearson test, L1 and E2 antibody levels correlated significantly at 2- and 12-month visits: *r* = .138 (*P* = .034) and *r* = 0.145 (*P* = .017), respectively. Significant correlations were also found between L1 and E4 antibody levels at the 6- and 12-month visits: *r* = 0.176 (*P* = .004) and *r* = 0.157 (*P* = .01). Only 1 significant correlation was found between L1 and E6 antibody levels, at the 6-month follow-up visit: *r* = 0.174 (*P* = .005). At 12 months, L1 and E7 antibody levels were significantly correlated: *r* = 0.121 (*P* = .046).

**Table 2.** Levels of Human Papillomavirus Type 6 L1, E2, E4, E6, and E7 Antibodies at Each Follow-up Visit of the Children

HPV-6 Protein	Follow-up Visit					
	1 mo (n = 232)	2 mo (n = 239^a^)	6 mo (n = 263)	12 mo (n = 272)	24 mo (n = 250)	36 mo (n = 243)
L1	242.49 (422.19)	151.33 (258.76)	144.93 (398.17)	275.84 (604.29)	390.12 (677.41)	245.55 (404.09)
E2	19.00 (50.66)	**17.32 (41.16)**	26.00 (38.83)	**45.15 (51.57)**	67.71 (155.90)	80.91 (139.45)
E4	11.02 (15.10)	11.17 (17.49)	**18.61 (25.89)**	**30.43 (33.82)**	37.21 (43.24)	38.65 (31.86)
E6	4.71 (8.86)	5.95 (12.73)	**15.39 (26.00)**	26.79 (30.54)	30.85 (30.71)	38.86 (114.32)
E7	2.65 (6.95)	2.51 (5.96)	5.89 (13.03)	**11.88 (16.23)**	15.96 (21.01)	17.25 (62.63)

Data are presented as mean (standard deviation). Bold values represent E2, E4, E6, or E7 antibody levels that are significantly correlated (Pearson) with L1 antibody levels (details in the Results section).

Abbreviations: HPV-6, human papillomavirus type 6; n, number of children.

^a^Only 237 children for E2 and E4.

The proportions of infants/children with positive antibody titers to HPV-6 L1 and E proteins at each follow-up visit are depicted in Table 3. Of the 232 neonates sampled at the 1-month visit, 31.5% were seropositive to L1, 3.9% to E2, and 0.4% to E4. The proportion of HPV-6 L1–seropositive children remained high throughout the entire follow-up, showing some fluctuation between 18% (at 6 months) and 37% (at 24 months). At all visits, seroprevalence to HPV-6 E2, E4, E6, and E7 was always substantially lower than that of L1. Among the E proteins, seropositivity to E2 exceeded all the others, ranging from 2.5% (2-month visit) to 17.3% (36-month visit). Table 3 lists the median times (and range) for seroconversion observed for the individual HPV-6 protein antibodies during the follow-up. Altogether, 159, 70, 32, 19, and 13 children seroconverted to HPV-6 L1, E2, E4, E6, and E7, respectively. Time to seroconversion was shortest for L1 (median, 11.8 months [range, 0.9–38.1 months]). Seroconversion to E2, E4, and E6 took slightly longer: 23.7, 23.2, and 23.4 months, respectively. The few events (n = 13) of seroconversion to HPV-6 E7 were detected much later, with a median time of 35.4 months (range, 5.8–37.1 months).

**Table 3.** Proportion of Children Seropositive to Human Papillomavirus Type 6 L1, E2, E4, E6, and E7 Proteins at Each Follow-up Visit, and Time to Seroconversion

HPV-6	Follow-up Visit						Seroconversion	
	1 mo (n = 232)	2 mo (n = 239^a^)	6 mo (n = 263)	12 mo (n = 272)	24 mo (n = 250)	36 mo (n = 243)	No.	Median Time, mo (Range)
L1	31.5 (73)	18.8 (45)	17.8 (47)	25.0 (68)	37.2 (93)	31.3 (76)	159	11.8 (0.9–38.1)
E2	3.9 (9)	2.5 (6)	3.4 (9)	9.2 (25)	12.4 (31)	17.3 (42)	70	23.7 (1.0–42.2)
E4	0.4 (1)	0.8 (2)	2.3 (6)	4.8 (13)	8.0 (20)	5.3 (13)	32	23.2 (5.2–42.2)
E6	0.0 (0)	0.4 (1)	1.5 (4)	3.7 (10)	6.0 (15)	4.1 (10)	19	23.4 (5.3–36.0)
E7	0.0 (0)	0.0 (0)	0.4 (1)	0.4 (1)	1.6 (4)	3.3 (8)	13	35.4 (5.8–37.1)

Data are presented as proportion (%) of seropositive children. Cut-off for L1 seropositivity was median fluorescence intensity (MFI) ≥200. Cut-off for E2, E4, E6, and E7 seropositivity was MFI ≥100.

Abbreviation: HPV-6, human papillomavirus type 6.

^a^Only 237 children for E2 and E4.

## DISCUSSION

We have previously published data on the dynamics of antibody response to L1 protein of the high-risk and low-risk HPV genotypes in mothers and their newborn in the FFHPV cohort, and its results confirmed that IgG antibodies to HPV-6, HPV-11, HPV-16, HPV-18, and HPV-45 L1 were vertically transferred from the mother to her offspring. There was a close concordance between maternal and newborn HPV antibody levels during the first 6 months after delivery. This coincides with the well-known natural decay of immunoglobulin molecules. It is noteworthy that none of the mothers had received prophylactic HPV vaccination before entering the study, which means that the serological responses to different HPV genotypes are all resulting from a naturally acquired HPV infection [22]. In general, the results of the FFHPV cohort concerning HPV L1 antibody dynamics among mothers and their offspring are concordant with those of the few previously published studies [4, 19–22, 28]. These data confirm a close HPV-type-specific concordance between maternal and newborn antibodies, irrespective of the methods used for blood sampling of the newborn (cord blood, dried blood spot, or serum) or for HPV antibody testing [19, 29, 30].

In this study, we were particularly interested in assessing whether these E-protein antibodies also show a transplacental transfer from the mother during fetal time, and whether a seroconversion to these HPV E proteins does occur during the infant's first 3 years of life. The low-risk HPV genotype HPV-6 was selected as the target of this study because this HPV type is clinically important, particularly in childhood, as it causes the vast majority of juvenile-onset laryngeal papillomas, which are difficult to treat in neonates and young children. Another argument favoring the selection of HPV-6 is that previous studies on serological response to HPV-6 E proteins in infants and young children are lacking, as most of the published studies have focused only on the high-risk types HPV-16 and HPV-18. In this longitudinal setting, the infant's antibody levels to E2, E4, E6, and E7 at the age of 1 month correlated very closely to the respective antibody levels of the mother in samples taken a few weeks before delivery. Second, the MFI levels of antibodies to all of these E proteins were much lower than the levels of IgG L1 antibodies. Third, as with L1 antibodies, these HPV-6 E protein antibody levels increased steadily with age up to the end of the 3-year follow-up. Fourth, there was an interesting correlation of infants’ HPV-6 L1 antibodies to HPV-6 E4 and E6 at the 6-month visit and, similarly, at 12 months, HPV-6 L1 antibody levels correlated significantly with the levels of HPV-6 E2, E4, and E7 antibodies.

We identified 3 early studies where antibodies to HPV E proteins have been studied in healthy children aged 1 to 10 years [31–33]. One study reported E4 antibodies in up to 30% of the girls aged between 1 and 10 years [31]. Contradictory to this, another study of 46 girls with the same age distribution did not disclose E4 antibodies in any of them [32]. An obvious reason to this discrepancy could be the differences in the E4 protein used as an antigen (truncated vs complete). One study analyzed HPV-16 E4 and E7 antibodies with the peptide-based enzyme-linked immunosorbent assay among healthy individuals (1–95 years of age), patients with HPV infection, and from patients who were at high risk for HPV infection, and its results demonstrated that the prevalence of anti-E7 antibodies increased with age, although the overall prevalence in the adult population was low (10.4%) [33]. The pattern of age-specific prevalence of anti-E4 antibodies was totally different, being low in the adult population (1.1%) and exceeding 20% among children and teenagers [33]. In addition, HPV-16 E4 antibodies were significantly associated with anti-E7 positivity among children, indicating that HPV-16 infection can frequently occur early in life [33].

In our study, we found a significant association between L1 antibodies and those to E2, E4, and E7 proteins among children aged 1 year. IgG antibody levels to HPV-6 L1 and to all of the E proteins also increased until the age of 2 years. It is important to note that these significant alterations between the L and E protein antibodies were observed at the 6-month and at the 12-month visits when maternal antibodies had already vanished and infants had developed their own immune response against HPV-6, which suggests that HPV-6 infection occurs in early life. The observed low antibody titers during the early months could be explained by low viral loads and transient expression of E proteins not sufficient to elicit an antibody production that reaches the MFI cut-off used for seropositivity. At the 12-month and 36-month follow-up visits, filled questionnaires were received concerning 266 and 203 children, respectively. At the 12-month follow-up visit, 1, 2, and 3 children had warts in feet, body, and hands, respectively. At the 36-month follow-up visit, there were warts in feet, body, and hands of the children in 2, 1, and 6 cases, respectively. No oral warty lesions were reported in any of the children at the age of 1 year or 3 years. Clearly, generation of antibodies in quantities sufficient to exceed the cut-off to these proteins may require a continued expression of early HPV gene products upon the primary HPV-6 infection, which is likely to occur in most cases between 1 and 3 years of age. Of particular interest is the intimate association between HPV-6 L1 to E2, E4, and E7 antibodies at 12 months, which precedes the emergence of seroconversion to all these proteins. It can be speculated that this concomitant appearance of both E and L1 antibodies coincides with an initiation of the first active production of mature HPV particles by the infants, to be manifested as an active seroconversion to the HPV-6 E proteins some months afterward. Apart from an acquired primary HPV-6 infection shortly before, one cannot rule out a possible reactivation of a latent HPV-6 infection acquired vertically from the mother.

A confirmed seroconversion to HPV L and E proteins during the first years of life is an indicator of an early exposure to HPV infection. In our study, seroconversion to L1 protein did occur earlier than seroconversion to E proteins (median time, 12 vs 23–35 months). Seropositivity of these very young children in our cohort indicates a previous exposure to HPV infection, most likely acquired by vertical transmission from the mother. Considering our findings, serum IgG antibodies against the E proteins might be useful candidates to monitor early HPV infections in children. However, as generally acknowledged, all HPV serology studies suffer from basic handicaps: Not all HPV infections induce seroconversion, and the stability of HPV antibodies over time may be variable [34, 35]. In addition, there is no gold standard or commercial method for analyzing HPV antibodies, which is yet another major limitation, particularly regarding the mutual comparison of HPV serology studies. Furthermore, for this study a limitation is that for L1 no other assays or neutralization were done due to the absence of these assays at the time of these antibody analyses.

Taken together, our study demonstrates that maternal antibodies against HPV-6 early and late proteins are transferred to a mother’s offspring. Furthermore, our results provide convincing evidence that seroconversion against HPV-6 L1, E2, E4, E6, and E7 does occur in early childhood. Further studies are needed to assess the role of maternal antibodies in protecting their offspring against primary HPV infection.

## Notes

***Financial support.*** This work was supported by the Finnish Medical Foundation (to K. L. and H. S.); the Sigrid Juselius Foundation (to K. L.); Research Council Finland (to K. L.); and the Emil Aaltonen Foundation (to H. S.).
